# Tissue Factor Expression in the Symptomatic Carotid Plaque

**DOI:** 10.4021/jocmr2009.07.1250

**Published:** 2009-07-23

**Authors:** Hani Abdul-Jabar, Abbas Rashid, Amir Sadri, Trevor Paes

**Affiliations:** a Department of Vascular Surgery, The Hillingdon Hospital, Pield Heath Road, Uxbridge, Middlesex UB8 3NN, UK

## Abstract

**Background:**

The aims of this study were to identify that the differences in the natural history of patients with symptomatic and asymptomatic carotid stenosis may be reflected in differences in the expression of procoagulant protein factors.

**Methods:**

Carotid artery plaques were obtained from 33 symptomatic and 4 asymptomatic patients with internal carotid artery stenosis of greater than 70%. These plaques were stained with monoclonal antibody against human tissue factor. Areas of staining for the cap and core were analysed using the analySIS computer programme.

**Results:**

There were 37 patients, of whom 27 were male with a mean age 69.3 years and a range of 53 to 83 years. Statistical analysis using non-parametric tests revealed a significant increase in the area of positive staining for tissue factor in plaques taken from symptomatic patients when compared to those who were asymptomatic (P = 0001). Within the symptomatic patients group there was significantly increased tissue factor in the plaque core of those who were the most recently symptomatic (P = 0.003).

**Conclusions:**

The unstable carotid artery plaque is associated with significantly increased tissue factor expression in the cap and core. Plaques from the most recently symptomatic patients have significantly more tissue factor in the core and this may represent part of the mechanism responsible for plaque destabilisation. More research is needed in this important area.

**Keywords:**

Tissue Factor; Carotid stenosis; Stroke; Plaque stability

## Introduction

Stroke is the third commonest cause of death in the UK after coronary disease and cancer, and the principal cause of neurological disability [1]. The annual UK incidence of stroke is 2:1000 and each year 125000 patients will suffer their first stroke [2]. Half of strokes affect patients over 75 years and only 25% occur in patients under the age of 65 [1]. Stroke patients in the UK utilise 10% of hospital bed-days and 5% of annual health expenditure [3]. Stroke mortality within the UK decreased by up to 20% over the last 30 years [4]. However, because of an increase in the ageing population, the overall incidence of stroke could increase by up to 30% by 2033 [5]. The vast majority of strokes are ischaemic, of these 80% affect the carotid territory.

The commonest cause of ischaemic stroke is thromboembolism of the internal carotid artery (ICA). Stenoses develop at the origin of the ICA because this is a complex region with haemodynamic phenomena compromising low shear stress, flow stasis and flow separation that predispose to atherosclerotic plaque formation. Should the plaque undergo acute change (rupture, ulceration, or intraplaque haemorrhage), the inner core of thrombogenic subendothelial collagen is exposed and this predisposes towards the formation of thrombus and the onset of symptoms [6].

### Carotid artery disease

The carotid artery plaque is a dynamic structure. It may be stable and unlikely to produce symptomatic embolization and carotid occlusion or conversely, while not necessarily being any more stenotic, unstable and at high risk of producing symptomatic embolization or carotid occlusion [7].

#### Mechanism of stroke in symptomatic carotid artery disease

The majority of strokes are due to embolization from an atherosclerotic plaque or acute occlusion of the carotid artery and propagation of thrombus distally [7], however a small number of strokes can be attributed to hypoperfusion [8]. Low flow alone is not usually sufficient to cause ischaemic stroke distal to symptomatic carotid stenosis. Post-stenotic narrowing may be protective because low blood flow distal to the stenosis is insufficient to carry emboli to the brain [9].

#### Relationship between presenting symptoms and stroke

Risk factors for an ischaemic stroke include: sex, increasing age, smoking, hypertension, ischaemic heart disease, cardio-embolic source, peripheral vascular disease and diabetes [6]. Presenting symptoms significantly predict outcome after carotid endarterectomy (CEA). Patients with amaurosis fugax (AF) have a significantly better survival than those with transient ischaemic attacks (TIAs), transient strokes or progressive strokes [10]. A history of crescendo TIAs and being female are associated with an increased incidence of perioperative death and stroke within 30 days of the operation. Deaths between 1 and 36 months are associated with both ischaemic heart disease and diabetes in both sexes [10].

### Carotid atherosclerotic plaque morphology

Angiographic plaque surface morphology is used along with the degree of carotid stenosis to identify patients most likely to benefit from CEA and other preventive treatment [11]. MRI angiography, CT scans and colour coded duplex ultrasonography are now very reliable diagnostic techniques and have largely superseded routine arterial angiography for surgical planning [12]. Biasi et al recently showed a relationship between plaque echogenicity and stroke risk. His study demonstrated that the grey scale median of a plaque, measured using a computerised method is associated with the presence or absence of CT-brain infarction. Plaques with high grey scale median (echogenic) were associated with an 11% incidence of CT-brain infarction, whereby plaques with low grey scale median (echolucent) were associated with a 55% incidence of CT-brain infarction [13]. Previous studies have already suggested that the presence of CT-brain infarcts is a risk factor for subsequent stroke and death [14-17].

#### Cellular biology and plaque rupture

Previous histological examinations have demonstrated subtle differences in the characteristics of atherosclerotic plaque removed from symptomatic patients. In symptomatic patients inflammation is more common, with greater number of macrophages and T cells detected in the cap of the symptomatic plaque [18-20]. The necrotic core is also placed nearer to the fibrous cap and the minimum cap thickness is less [7].

Smooth muscle cells lay down collagen, the principal connective tissue component of the fibrous cap. Collagen breakdown is dependant on the balance between proteolytic enzymes; matrix metallaproteinases (MMPs) and their inhibitors; tissue inhibitors of metalloproteinases (TIMPs). High levels of MMPs have been demonstrated at the site of the inflammatory infiltrate in the fibrous cap [21], smooth muscle cell apoptosis has also been demonstrated in unstable carotid plaques [22].

T-cells secrete CD-40 and interleukin-1 (IL-1), which activate macrophages which in turn stimulate the apoptotic activity of MMPs resulting in collagen degradation [7].

#### Plaque thrombogenicity

On plaque rupture, exposure of the necrotic core to circulation promotes thrombosis. This appears to be an important mechanism of plaque progression, in addition to embolization. Increased expression of tissue factor, the most important stimulant of the extrinsic clotting cascade, has been demonstrated in plaques from patients with unstable angina or myocardial infarction [23]. In an animal model, plaque rupture is associated with increased tissue factor production from circulating monocytes, which is reduced by treatment with a nitrous oxide precursor [24].

### Tissue factor (TF)

TF is a 47-kD membrane bound, low weight molecular glyco-protein essential for the activation of the extrinsic coagulation pathway. TF receptor is a member of the class 2 cytokine receptor super-family. Growth factors and cytokines present in vascular smooth muscle cells and macrophages regulate the TF expression [25].

TF complexes with factors VII and VIIa, permitting enzymatic activation of factors X and IX, the substrates for factor VIIa [26], and ultimately leading to the generation of thrombin [26]. TF is strongly induced in activated inflammatory macrophages and T cells. By its ability to bind factor VIIa; TF directly activates the coagulation cascade. Therefore, TF is a candidate molecule linking plaque inflammation with arterial thromboembolism. In the present study, we carried out an immunocytochemical analysis of TF expression in endarterectomy specimens from 37 patients undergoing CEA for high-grade ICA stenosis and addressed the relationship of TF expression to clinical features of plaque instability.

## Subjects and Methods

### Patients

The study included 37 surgical in-patients enlisted to undergo CEA for high-grade ICA stenosis (≥ 70% luminal narrowing). The study was approved by the local ethics review committee and performed in accordance with institutional guidelines. Informed consent was obtained from all patients. Baseline characteristics of the study population are provided in Table 1. The degree of stenosis was determined by Standardised Imaging and Doppler Criteria for Cerebrovascualr Diagnosis using Duplex Sonography [27]. During surgery intra-venous heparin was given to all patients as an anti-coagulant.

**Table 1:** Clinical features of symptomatic and asymptomatic patients with high-grade ICA stenosis (≥70%)

	Age (mean ± SD)	Sex (Male)
All Patients (n=37)	69 ± 7	27
Asymptomatic (n=4)	72 ± 4	1
Symptomatic (n=33)	69 ± 9	26
Time Since Symptoms	Number	Sex (Male)
Asymptomatic	4	1
1-6 Months	17	16
< 1 Month	16	10
Type of Symptoms	Number	Sex (Male)
Asymptomatic	4	1
TIA/AF	26	5
Stroke	7	21

### Histological procedure and immunocytochemistry

After longitudinal arteriotomy, the carotid atherosclerotic plaque was excised en bloc (routine endarterectomy), fixed immediately in buffered formalin, decalcified, and transversely sectioned at 5-mm intervals [28]. Each 5-mm tissue bloc was embedded separately into paraffin. For immunocytochemistry, 3-mm sections were mounted onto gelatine-coated slides. After deparaffinization, sections were incubated with monoclonal antibody (mAb) against human tissue factor (No.4508, American Diagnostica Inc) at 1:200 dilution, followed by goat anti-mouse (Vector Laboratories) and the ABC ELITE kit reagents (Vector) with diaminobenzidine as a substrate. For antigen retrieval all sections were microwaved in 10mmol/L sodium citrate buffer, pH 6.0, for 10 minutes before staining.

Absence of tissue factor antibody was regarded as a negative control, while a positive control was the presence of tissue factor antibody in a human colon cancer tissue. Mayers hematoxilyn was used to counter-stain all the sections to enable the histopathologist to review the slides.

### Quantification

The analySISTM computer program was used to calculate the total area of positive TF staining within the cap and core of each specimen.

### Statistical Analysis

Paired sample T-tests were not used to analyse the data, as it was not normally distributed (v values of the Shapiro-Francia W normality test were outside the range of 2.0-2.8, v value of cap = 3.6, v value of core = 1.8). The non-parametric Mann-Whitney U test was used to investigate the relationship between the extent of TF expression (median TF+ section area in the cap and core of each plaque) and the occurrence of ischaemic symptoms. Kruskal-Wallis non-parametric test was used to study the relationship between TF expression (median TF+ section area in the cap and core of each plaque) and the time since symptoms. P values of < 0.05 were considered indicative of statistically significant findings. Measurement errors were calculated to account for the natural variation of the measurement process using the repeatability method [29]; values calculated were not significant (mean difference = 915.4, mean within-subject standard deviation = 647.3).

## Results

**Figure 1:**
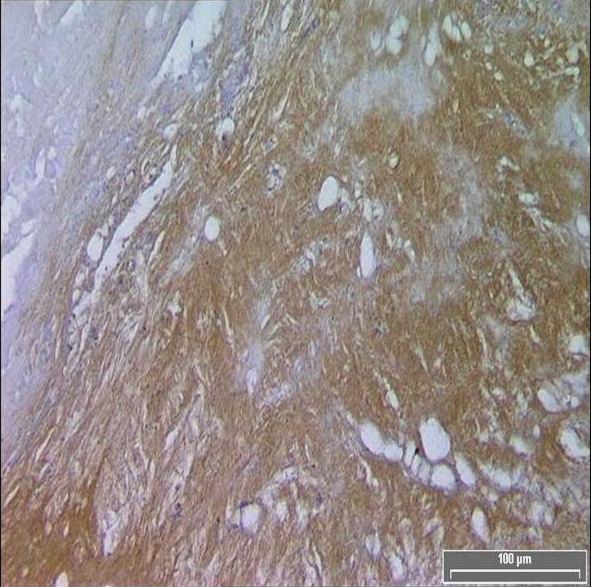
Diffuse TF immuno-reactivity in the core of a symptomatic patient.

**Figure 2:**
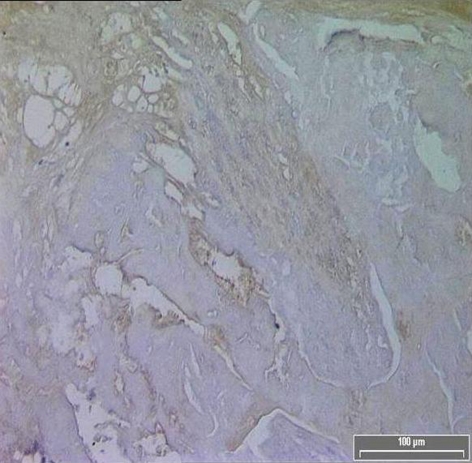
Diminished TF immuno-reactivity in the core of an asymptomatic patient.

**Figure 3:**
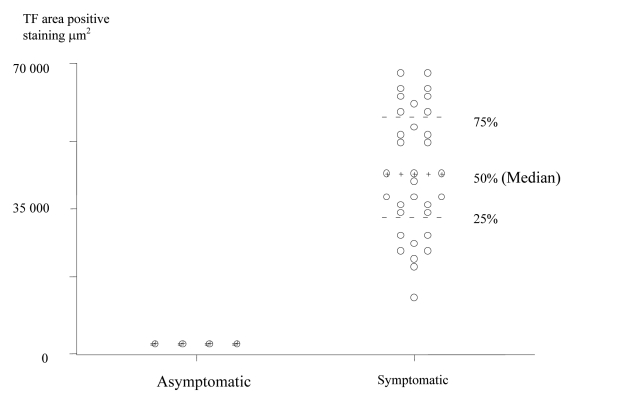
Scatter Plot of TF positive staining area in the Plaque Cores of Asymptomatic and Symptomatic Patients (units are expressed in μm^2^).

**Figure 4:**
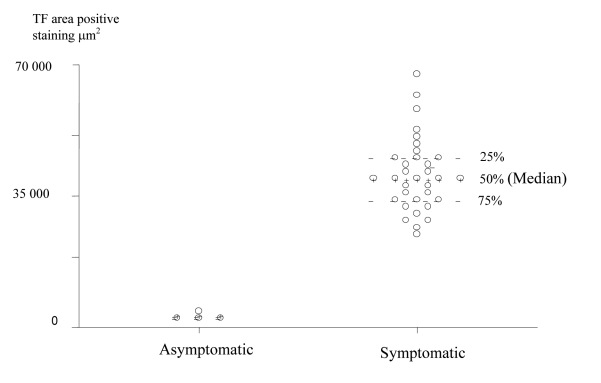
Scatter Plot of TF positive staining area in the Plaque Caps of Asymptomatic and Symptomatic Patients (units are expressed in μm^2^).

TF expression was found in all plaques, however there was a significant difference in the area of positive staining in plaques from symptomatic (Fig. 1) and asymptomatic (Fig. 2) patients. The resulting P values (Table 2) correspond to Mann-Whitney U non-parametric test with units of mm2 expressing the median and the interquartile range. (Fig. 3, 4)

**Table 2:** TF positive staining area in asymptomatic and symptomatic patients

	Asymptomatic	Symptomatic	P Value
TF Cap	379.1 [301.4-703.2]	37296.2 [32067.6-43648.7]	0.0010
TF Core	399.8 [282.2-710.4]	52689.2 [39798.5-70827.0]	0.0010

**Figure 5:**
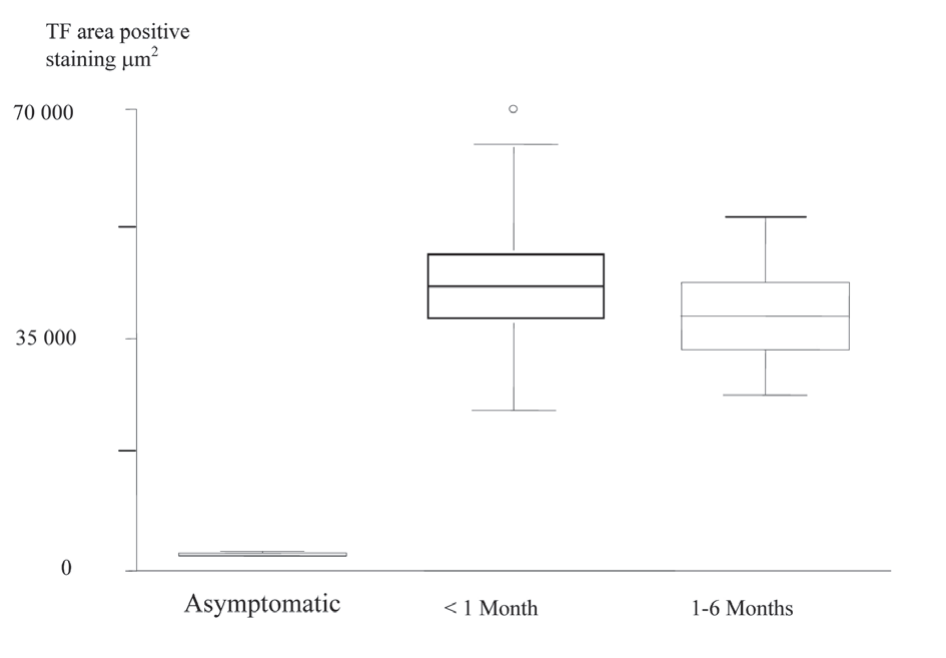
Box and Whisker plots, which show, increased TF Expression in the Plaque Cores of the most recently Symptomatic Patients.

The patients were then divided into groups according to the length of time between the last acute event and the CEA. Using the Kruskal-Wallis non-parametric test (Table 3); the expression of TF in the core was shown to be significantly increased in plaques from the most recently symptomatic patients. (Fig. 5).

**Table 3:** Increased TF positive staining area in the plaque cores of the most recently symptomatic patients

	Asymptomatic	< 1 Month	1-6 Months	P Value
TF Core	399.8 [282.2-710.4]	64386.2 [38320.6-75970.5]	46286.7 [39698.5-63462.7]	0.0030

## Discussion

In this study we have shown that in the most recently symptomatic carotid artery plaque there is an increased expression of TF when compared to the asymptomatic carotid artery plaque. Most TF immuno-reactivity was localised diffusely in the acellular necrotic core and in regions within the atheromas fibrous cap. Our data therefore strongly suggest that TF induction at various regions of the plaque may play an important role in the destabilisation of high-grade ICA stenosis.

The mechanism responsible for thrombus formation associated with ruptured atherosclerotic plaques is not well understood. One widely held view is that rupture or fissuring of an atherosclerotic plaque results in exposure of collagen and other extracellular proteins to blood-borne platelets, which adhere to these elements via specific receptors and become activated [30]. Activated platelets release ADP and thrombin, bind fibrinogen, and activate other platelets, thereby producing local thrombosis in and on the arterial wall [25].

Exposure of TF activity to coagulation factors may either occur directly at the intimal surface or may result from plaque rupture leading to the release of macrophage-bound or extracellular material from deeper parts of the plaque to the blood stream [31]. The rupture of complicated plaques has been suggested to be due to the expression of MMPs that degrade extracellular matrix components and thereby weaken the fibrous cap [21, 32]. A recent study by Loftus et al [33] showed a correlation between MMP-9 expression and carotid plaque destabilisation. Thus, it is an intriguing hypothesis that the concerted action of MMP-9 and TF may be a key mechanism of plaque destabilisation in cerebrovascular disease patients who are at high risk of stroke.

TF pathway inhibitor (TFPI) is found in vascular endothelium and smooth muscle cells as well as in platelets, blood monocytes, and macrophages [34, 35]. It provides physiological inhibition of TF-initiated coagulation by binding to factors Xa and the TF-factor VIIa complex in a 2-step process. In atherosclerotic carotid arteries, TF expression is abundant, whereas TFPI expression is limited in up to 30% of plaques, resulting in predominant TF activity [36].

Evidence also suggests that TF may have a variety of other actions in addition to initiating thrombin formation. For example, TF: VIIa induces the activation of mitogen-activated protein kinase (MAP-Kinase) in a baby hamster kidney [37] and evokes intracellular Ca+2 mobilisation in human endothelial cells [38], which may lead to cell proliferation.

Human atherosclerotic plaque tissue from CEA specimens express TF messenger-RNA and protein in a few intimal smooth muscle cells, as well as in a few macrophage-like cells adjacent to cholesterol clefts [25]. Wilcox et al [39] showed that TF antigen seemed to occur extracellularly. Annex et al [40] demonstrated antigenic TF in coronary atherectomy specimens but, as with previous immunohistochemical studies, TF was seen in only approximately one third of specimens. When using digoxigenin labelled factors VIIa and X, Thiruvikraman et al [25] showed diffuse TF activity both intracellularly (endothelial cells, smooth muscle cells, and macrophages) and extracellularly (lipid-rich core and fibrous matrix) in virtually all human atherosclerotic plaques taken from various sites.

A potential limitation of our study arises from the fact that the sensitive immuno-histochemical staining procedure used for the detection of TF antigen does not allow direct conclusions with respect to the actual presence of TF bioactivity. However in a comparative study, Jander et al [31] used a similar TF-specific antibody for the in-situ detection of TF and observed essentially identical staining patterns with a similar type of detection reagent; TF specific antibody bound to the atheromas acellular lipid core. It is therefore likely that the TF immuno-reactivity detected in our study indeed reflects TF binding activity for its physiologically relevant ligands. However, additional studies using TF bioassays will be necessary to definitively clarify the role of TF in ICA plaque destabilisation.
